# MicroRNAs’ Impact on Heart Diseases

**DOI:** 10.3390/ijms26125566

**Published:** 2025-06-10

**Authors:** Marco Antonio Cordeiro, Ana Elisa T. S. de Carvalho, Regina Celia Spadari

**Affiliations:** Laboratory of Stress Biology, Department of Biosciences, Health and Society Institute, Campus Baixada Santista, Federal University of Sao Paulo (UNIFESP), Santos 11020-015, SP, Brazil; m.santos08@unifesp.br (M.A.C.); aetscarvalho@unifesp.br (A.E.T.S.d.C.)

**Keywords:** cardiovascular diseases (CVDs), microRNAs (miRNAs), heart, epigenetics mechanisms

## Abstract

Cardiovascular diseases (CVDs) are the most prevalent cause of global mortality, highlighting the importance of understanding their molecular bases. Recently, small non-coding RNAs (miRNAS) were shown to affect messenger RNA (mRNA) stability, either by inhibiting translation or by causing degradation through base pairing with mRNAs, being negative regulators of protein translation. Moreover, miRNAs modulate many signaling pathways and cellular processes, including cell-to-cell communication. In the cardiovascular system, miRNAs control functions in cardiomyocytes, endothelial cells, smooth muscle cells, and fibroblasts. Because miRNA expression was detected in the blood of patients with various cardiovascular diseases, they are considered attractive candidates for noninvasive biomarkers. This study reviews the literature on the role played by miRNAs in heart development and diseases. The findings suggest that miRNA regulation may offer new perspectives for therapeutic interventions in heart diseases.

## 1. Introduction

The cardiovascular system (CVS) transports oxygen, nutrients, endocrine factors, and immune cells throughout the body, removes metabolic waste, and participates in thermoregulation. The heart is the component of the CVS that keeps the blood flowing so that its functions can be accomplished, and cardiovascular diseases are the leading cause of death worldwide. Estimates indicate that around 19.8 million people die every year due to cardiovascular diseases [1,2]. Despite advances in the prevention and treatment of those diseases, there is still much to be discovered regarding their underlying molecular mechanisms.

According to the Mendelian theory, an organism’s set of phenotypic characteristics is essentially determined by its genotype, so that the gene encoded from nuclear DNA is transcribed into RNA, which, in turn, is translated into a protein specific to each cell. This mechanism of gene expression is highly regulated, with it being possible to modulate the expression of genes in specific tissues and cells without altering the genome. The reprogramming of gene expression, with hyper- or hypo- regulation of a set of genes, is a critical process in the maintenance of homeostasis and functional modulation of tissues. Its deregulation can trigger pathologies.

Nevertheless, more recent evidence shows the existence of changes in expressed proteins without corresponding changes in their respective genes [3,4,5]. The mechanisms involved in those modifications are described in the literature as epigenetic factors, the main ones being DNA methylation reactions, histone modification, and non-coding RNA (ncRNA) synthesis [3,5,6].

ncRNA, which corresponds to 80% of the 3 billion base pairs in the human genome [7], is now recognized as an essential part of the mechanisms regulating gene expression [7,8,9]. In recent years, there has been growing interest in the function of small ncRNA called microRNAs (miRNAs), mainly because some studies have shown that they may be related to cardiovascular diseases [10,11]. About 2500 miRNAs have been identified in the human genome [12], with the number of functional miRNAs ranging from 556 (mirGene DB 2.0) [13] to 758 [13,14], and up to 150 miRNAs play a critical role in the cardiovascular system. Of those, 30 to 35 miRNAs have been comprehensively analyzed in experimental models in vivo [15].

MicroRNAs were shown to affect messenger RNA (mRNA) stability, either by inhibiting translation or by causing degradation through base pairing with mRNAs, being negative regulators of protein translation, thus playing fundamental roles in regulating cellular function and cell-to-cell communication [16,17]. Additionally, miRNAs interact with long non-coding RNAs (lncRNAs), circular RNAs (circRNAs), and pseudogenes to either induce miRNA suppression or increase cellular competition for miRNA binding sites [18,19,20,21].

As a result of their role in various biological systems [16] and stability in human fluids, miRNAs are considered to be putative biomarkers for diagnosis, prognosis, and treatment of many diseases [22,23]. In the cardiovascular system, miRNAs control functions of cardiomyocytes, such as growth and contractility, endothelial cells, smooth muscle cells, and fibroblasts [24]. miRNAs also control the cardiac rhythm [25,26,27], and their expression is altered in the blood of patients with various cardiovascular diseases [28,29].

Because each mRNA molecule may be regulated by multiple miRNAs, a single miRNA may influence the expression of hundreds of genes [30]. Many miRNAs are tissue-specific, while others are ubiquitously expressed [31,32,33]. Both transcriptional and post-transcriptional regulation of miRNA precursors within the cell determine their pattern of distribution. All known sequences of miRNAs are available in the database on the website https://www.mirbase.org.

Here we present a comprehensive literature review concerning the role played by miRNAs in heart development and heart diseases. To compile this bibliographic review, scientific articles indexed in the PubMed (https://pubmed.ncbi.nlm.nih.gov/) and Google Scholar databases, published between 2018 and 2024, were consulted. The following keywords were employed: microRNAs, cardiovascular diseases, heart diseases, and heart. Papers published in the above-mentioned period and containing the defined keywords were included, and those not related to the keywords or published outside of the defined period of time were not considered.

## 2. MicroRNA Biogenesis

**Figure 1:**
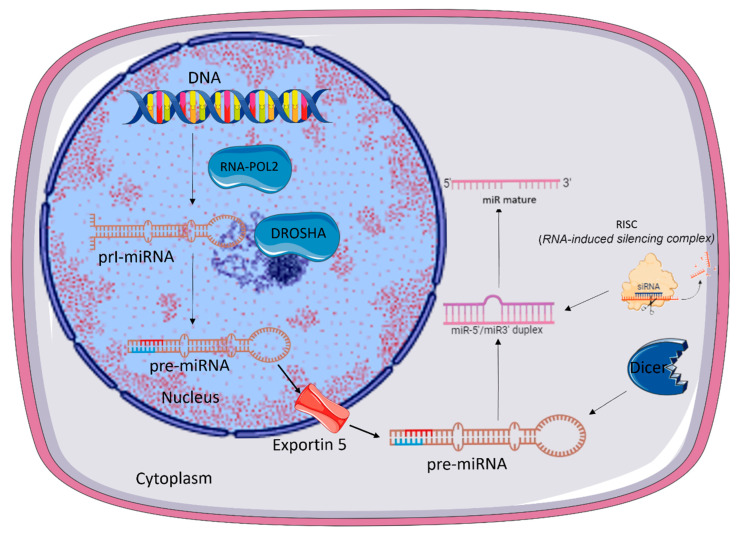
Canonical microRNA biogenesis. In the nucleus, primary transcripts (pri-miRNA) are processed by the Drosha enzyme, and a double-stranded RNA-binding protein forms a precursor microRNA (pre-miRNA), which is exported to the cytoplasm by exportin-5 located on the nuclear membrane. These pre-miRNAs are processed by Dicer, a nuclease, forming a double-stranded RNA that is incorporated into the RNA-induced silencing complex (RISC) and then separated into two strands. One of these strands is degraded, and the other remains associated with RISC, forming the mature microRNA (miR-).

The process of biogenesis of htta miRNA occurs in two parts: one in the nucleus and the other in the cytoplasm (Figure 1). In the nucleus, the transcription that generates primary transcripts (pri-microRNAs) starts [19], with a length of over 1000 nucleotides organized into three major domains: a 33- to 35-nucleotide-long stem terminal loop and two single-stranded RNA (ssRNA) segments flanking both ends [34,35].

Depending on the location of the miRNA gene within the genome, identified miRNAs are classified as intragenic or intergenic [18,28]. Intergenic-derived miRNAs are transcribed either by RNA polymerase II or III, come from non-coding regions of the DNA between genes, and have unique promoter regions [34,35,36,37]. By contrast, the genes of intragenic-derived miRNA are transcribed by RNA polymerase II. They are located within exons or introns of protein-coding genes and are co-expressed with their host gene [34,35,36].

The biogenesis of miRNA is classified into canonical and non-canonical pathways. In the canonical pathway (Figure 1), those pri-microRNAs are processed by the enzyme Drosha (RNAse III) and its cofactor DGCR8 (DiGeorge Syndrome Critical Region 8). The resulting precursor microRNA (pre-microRNA) is exported to the cytoplasm by exportin-5 through pores in the nuclear membrane [38]. In the cytoplasm, those pre-microRNAs are processed by Dicer, another RNAse [39], forming a double-stranded mature miRNA.

Before being separated into two strands, both strands can be loaded into the Argonaute (AGO) [40] and incorporated into the RNA-induced silencing complex (RISC). One of the strands is degraded, while the other remains associated with RISC, forming the mature microRNA [12,16,41,42].

The strand with lower thermodynamic stability is selected to be loaded into AGO and is deemed the guide strand [43]. The unloaded strand, the so-called passenger strand, is cleaved by AGO2 and degraded [44]. Mature microRNA is identified by the prefix “miR” while pre-microRNAs are identified by “mir-”.

The non-canonical miRNA biogenesis pathways can be grouped into Drosha/DGCR8-independent and Dicer-independent pathways [45].

Drosha/DGCR8-independent pathway may process the pre-miRNA splicing machinery from the intron of protein-coding gene [28,30]. Those microRNAs, called miRtrons, are correlated with the host gene expression based on their location in either the introns or splice site junctions. MiRtrons are continuously transported to the cytoplasm and processed by Dicer [46,47,48].

Conversely, in the Dicer-independent pathway miRNAs are processed by Drosha from endogenous short hairpin RNA (shRNA) transcripts [47]. AGO2 completes the maturation of these pre-miRNAs [47] since it promotes loading of the entire pre-miRNA into AGO2 and AGO2-dependent slicing of the 3p strand. The 3′-5′ trimming of the 5p strand completes their maturation [47].

Partially based on the thermodynamic stability at the 5′ ends of the miRNA duplex or the presence of a 5′ uridine (U) at the nucleotide position, the 5p or 3p strand is selected [47].

The seed region is essential for recognizing and binding to the target mRNA, determining which genes the miRNA can regulate. MicroRNAs with identical seed sequences belong to the same seed family and typically regulate a similar set of genes. For example, miR-133a and miR-133b share the same seed sequence, so they target many of the same biological pathways [47].

MicroRNAs can be organized into clusters, meaning that multiple microRNA genes are located close together in the genome and transcribed as a single polycistronic RNA. The miRNAs within a cluster generally have related functions and can act synergistically in regulating target genes [48].

## 3. Factors Modifying miRNAs

In the last thirty years, following the identification of the initial microRNA (miRNA), researchers have discovered thousands more, each essential for managing gene expression in multicellular life forms. Nonetheless, the complex relationships within the miRNA and mRNA regulatory framework are still not completely understood [49].

The levels and variety of miRNAs found in cells are precisely controlled in several phases, which encompass transcription, modifications after transcription, maturation, and breakdown. This management is vital for ensuring the proper functionality of miRNAs in influencing mRNA translation. Various DNA-binding proteins—like tumor suppressors such as BRCA1, transcription regulators like p53, and RNA helicases like DHX9—can affect how effectively and accurately the microprocessor complex operates during the early stages of miRNA processing. Furthermore, interactions between Dicer and certain conserved RNA-binding proteins, including TARBP2 (a component of the RISC-loading complex), are essential for the correct selection of strands in miRNA maturation [50,51].

Within the cellular setting, different RNA types and RNA-binding proteins vie for connections with the available miRNAs [52]. These include long non-coding RNAs (lncRNAs), pseudogenes, and circular RNAs (circRNAs), among others. This competition plays a significant role in determining how accessible and functionally potent miRNAs are [53,54]. Despite progress, existing tools and algorithms for forecasting miRNA targets do not adequately reflect the full complexity of these interactions. Therefore, precise quantitative evaluations of binding affinities, molecular levels, and stoichiometry between miRNAs and their targets are crucial for grasping the dynamics of regulatory frameworks [55,56].

The ultimate result of miRNA processing, which involves creating and stabilizing isomiRs, is affected by cell-specific accessory proteins that interact with the main components—namely the microprocessor complex, Dicer, and Argonaute (Ago) proteins.

Maintaining cellular balance relies on nonlinear interactions among various miRNAs, regulated by their relative quantities and accessibility [55,57]. Additionally, spatial and temporal considerations are crucial in shaping the regulatory environment of miRNAs. Importantly, miRNAs participate in intricate interactions with other RNA entities, such as lncRNAs, small nuclear RNAs (snRNAs), and circRNAs. Many of these RNA types possess several binding sites for miRNAs and act as competitive endogenous RNAs (ceRNAs), serving as “sponges” that draw miRNAs away from their mRNA targets [57]. This process reduces the local concentration of active miRNAs, thereby restricting their regulatory capabilities.

Consequently, in addition to the identity and sequence of miRNAs, their accessibility to bind with target transcripts becomes a vital aspect of cellular regulation. This added dimension of RNA–RNA interactions signifies a complex and evolving area that warrants further exploration.

## 4. MicroRNAs and Intercellular Communication

miRNAs were first discovered as acting inside cells by controlling gene expression at a post-transcriptional level. More recently, however, they have also been detected in circulating blood. MicroRNAs which migrate outside the cells play a role in intercellular communication [58,59]. Those miRNAs are called circulating miRNAs [60].

In order to be protected from digestion by RNAses [60], the circulating miRNAs are secreted in exosomes or extracellular vesicles (EVs) [61,62,63] or form complexes with proteins including Ago2 [50], nucleophosmin 1 (NPM 1), and high-density lipoprotein [64]. Unlike intracellular miRNAs, circulating miRNA shows remarkable stability and resistance to degradation by endogenous RNAse activity [65,66].

Exosomes containing miRNAs enter neighboring cells through endocytic uptake, membrane fusion, or integrating with specific receptors in the cell surface, thus affecting mRNA targets remotely from their origin [66,67]. The circulating miRNAs in exosomes can reflect the specific cell of origin of the exosome content, and the variation depends on the physiological and pathological condition [66]. Precisely because their content reflects their origin and pathophysiological state, exosomes and extracellular vesicles have been used as biomarkers for diagnostic and therapeutic strategies.

The main source of circulating miRNAs seems to be the adipose tissue, although they may be derived from many other tissues. Adipose-tissue-secreted extracellular vesicles containing miRNA may arise from different cells within the fat pad and may be differentially regulated by various stimuli [17].

Obesity changes the profile of exosomal miRNAs in mice [62]. Moreover, obesity-associated exosomal miRNAs are active players in the first stages of the metabolic syndrome characterized by development of glucose intolerance, dyslipidemia, and central obesity [68].

miRNAs are secreted by pancreatic islet cells along with secretion of insulin and glucagon [69]. In that case, miRNA would modulate insulin action in target tissues, while peripheral cells could send signals back to islets via extracellular vesicles containing miRNAs. MiR-122, miR-142–3p, miR-192, miR-222, and miR-378a were upregulated, and miR-138 and miR-221 were downregulated in obese patients and obese animals [69,70].

Circulating extracellular vesicles may also cross the ependymal layer and the blood–brain barrier (BBB), by a transcytosis mechanism, thus acting on the central nervous system [71,72]. Neurodegenerative diseases which alter BBB permeability could facilitate the exchange of circulating miRNAs from the brain to the blood and vice versa [70].

Communication between the different cell types that make up the heart, such as cardiomyocytes, fibroblasts, endothelial cells, and macrophages, has already been described as important for maintaining cardiac homeostasis [73,74]. This intercellular communication can occur through direct contact between cells, such as gap junctions, through the secretion of soluble factors, or extracellular vesicles [75].

In cardiac diseases, such as myocardial infarction, miRNA transfer by extracellular vesicles plays a key role in driving the healing and remodeling response [75]. They can modulate the phenotype and function of macrophages, which in turn modulate fibroblast proliferation and the inflammatory response [75]. EVs from ischemic cardiomyocytes can also stimulate endothelial cell angiogenesis by miRNA transfers, mainly miRNA-222 and miRNA-143 [76].

## 5. miRNAs in Cardiac Development and Diseases

Epigenetic mechanisms are present at all stages of cardiac development. Eighteen miRNA families account for approximately 90% of cardiac miRNAs, regulating the initial process of cardiac differentiation and cardiomyocyte proliferation [77,78]. miRNAs participate in cell cycling, cell proliferation, cellular aging, apoptosis, angiogenesis, autophagy, mitochondrial metabolism, hematopoiesis, and cardiovascular development. Circulating levels as well as the source of miRNAs are altered in cardiovascular diseases [79].

**Figure 2:**
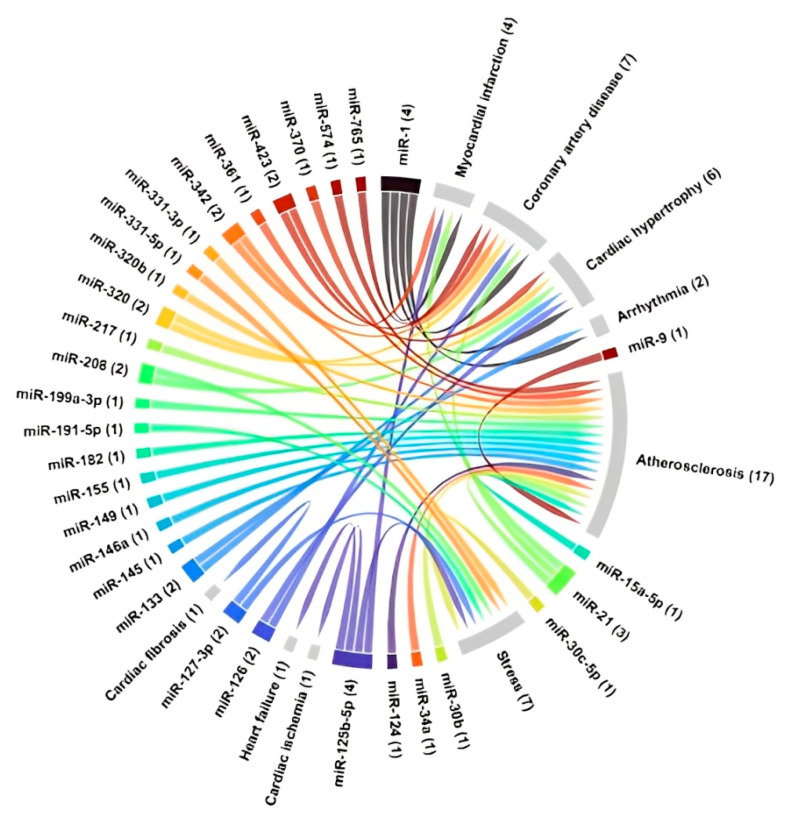
Chord diagram illustrating the associations between different RNAs and specific cardiac conditions, such as cardiac hypertrophy, myocardial infarction, and arrhythmia, highlighting the complexity of molecular interactions in the context of cardiovascular diseases. Each color represents one miRNA, and arrows indicate the linkage with cardiovascular disease. The numbers between parenthesis indicate the number of interactions between the disease and the microRNAs.

Altered expression of miRNAs has been described in cardiomyopathy, atrial fibrillation, hypertension, metabolic syndrome, and stroke, angiogenesis, inflammation, and cardiac remodeling, which are central to the development and progression of cardiovascular diseases [79,80,81,82,83]. The relation of some miRNAs and heart diseases are summarized in Figure 2 and in Table 1.

The first-described miRNAs were lin-4 and let7, both in *C. elegans* [84] and highly expressed in cells of the cardiovascular system [77,78,80,81,82] Exacerbated expression of let7 has been linked to cardiac fibrosis, myocardial infarction, cardiac hypertrophy, arrhythmia, atherosclerosis, and hypertension. Let7 also acts in cardiovascular differentiation from embryonic stem cells [77,83,84,85].

The regulatory potential of miRNAs in cardiac tissue homeostasis was demonstrated by the loss of a component of miRNA’s biogenesis machinery in an animal model, which was incompatible with proper cardiac function and maintenance of life. Mutant mice presented premature lethality, displaying dilated cardiomyopathy and heart failure [83], which shows the importance of miRNAs in the heart.

### 5.1. MiRNAs Involved in Cardiac Development and Function

The most abundant miRNAs expressed in myocardial tissue involved in the regulation of cardiomyocyte differentiation, in the early stages of heart development, are miR-1, miR-133a, miR-208, and miR-499 [84]. First, MiR-1 and miR-133 cooperatively promote mesoderm differentiation in embryonic stem cells. Then, they play opposite roles: miR-1 promotes and miR-133 inhibits differentiation of the mesoderm into cardiomyocytes [85].

MiR-1 regulates the expression of Irx5, which regulates the expression of potassium channel genes, thus determining the cardiac ventricular repolarization gradient [86]. Mir-1 also modulates Hand2 expression, which participates in the development of the outflow tract and right ventricle [84].

MiR-133 regulates genes responsible for differentiation and growth of cardiac and smooth muscle. MiR-133 also modulates Cyclin D2, which controls cardiomyocyte proliferation by acting on the phosphorylation of retinoblastoma protein in the G1 phase of the cell cycle [87]. MiR-208a, miR-208b, and miR-499 keep the contractile protein expression strictly under control, thus avoiding pathological cardiac remodeling [85,87,88]. MiR-15b controls the ATP level in cardiomyocytes by targeting Arl2, a component of the ADP/ATP exchanger in mitochondria [86]. MiR-195 controls numerous cell cycle genes; it is upregulated immediately after birth and halts cardiomyocyte proliferation. Overexpression of miR-195 results in ventricular septal defects (VSDs) and ventricular hypoplasia [86].

The miR-17~92 cluster consists of miR-17, miR-18a, miR-19a/b, miR-20a, and miR-92. MiR-17~92 transcription is activated by the bone morphogenetic protein (BMP) signaling pathway. Downregulation of Isl1 and Tbx1 in miR-17~92 promotes the development of the cardiac outflow tract and the differentiation of the second heart field (SHF) progenitors into right ventricle myocytes [89]. Deletion of miR17~92 leads to ventricular septal defects and lung hypoplasia, and consequently death. These effects are attributed in part to the upregulation of pro-apoptotic proteins like Bim, which is a target gene of this miRNA cluster [89].

Activity and expression of those miRNAs must be strictly regulated to ensure proper cardiac function. In contrast, their dysregulation is associated with the occurrence and progression of cardiac disease, including heart failure and its associated deleterious processes, such as arrhythmias, apoptosis, hypertrophy, fibrosis, and reverse remodeling [90].

**Table 1:** Expression of microRNAs alterations in heart diseases and stress.

Disease	Expression miRNAs	Reference
Myocardial infarction		
Up	miR-1, miR-133, miR208, miR208a, miR-352, miR-361, miR-423, miR-765	[91,92,93,94,95,96]
Down	miR-1, miR-126, miR133a, miR-133b, miR145, miR-149, mi-155, miR-17, miR-320, miR92a	[92,94,97,98]
Coronary artery disease/atherosclerosis		
Up	miR-1, miR-133, miR-155, miR182, miR-21, miR208a, miR-29, miR30b, miR-320b, miR-342, miR-370, miR-423, miR-451b, miR-574, miR-765, miR-9	[91,92,93,99,100]
Down	miR-126, miR-145, miR-149, miR-155, miR17, miR-320, miR92a	[92,93,98,101,102]
Cardiac ischemia		
Down	miR-125b	[103]
Cardiac fibrosis/hypertrophy		[104,105,106]
Up	miR-21, miR195, miR-208	
Heart failure		
Up	miR-29, miR-423, miR-499	[99,107,108]
Atrial fibrillation		
Up	miR-499	[107]

### 5.2. MiRNAs Involved in Cardiac Diseases

#### 5.2.1. Myocardial Infarction and Atherosclerosis

Acute myocardial infarction (MI) is defined as cardiomyocyte death due to acute imbalance between oxygen supply and demand, which causes a prolonged ischemia [109]. Ischemia/reperfusion injury associated with myocardial infarction leads to remodeling in the myocardium, which is regulated by various miRNAs.

One of the major contributors to myocardial infarction is atherosclerosis, which is a chronic inflammatory disease of the arterial wall involving insudation and retention of lipoproteins at sites of disturbed flow and accompanying dysfunctional endothelium [108,109]. Advanced coronary artery plaques are characterized by a lipid-rich/necrotic core associated with focal accumulations of inflammatory cells, covered by a fibrous cap. After the disruption of a vulnerable atherosclerotic plaque, its underlying thrombogenic core is exposed to the blood stream, resulting in thromboembolism (platelets adhere to the site of a rupture and release pro-inflammatory mediators, leading to thrombus formation) and subsequent acute coronary obstruction [89].

MiR-126, as well as circulating levels of miR-17, miR-92a, miR-155, and miR-145 are reduced in patients with atherosclerosis and coronary artery disease [92,97,100,106], while levels of miRNAs from cardiac muscle, mainly miR-133a and miR-208a, are higher [83]. Furthermore, expression levels of circulating miR-423, miR-320, miR-765, miR-149, miR-21, miR-126, miR-342, and miR-1 are altered in stable and unstable coronary artery disease and myocardial infarction patients [94,97].

Progression of atherosclerosis is related to several miRNAs, including miR-15a-5p, miR-199a-3p, miR-34a, miR-146a, and miR-217. Those miRNAs are also involved in cellular senescence, endothelial dysfunction, and inflammation, whereas miR-342-5p protects against endothelial cellular injury during atherosclerosis [89]. MiR-9, miR-21, mir-23a, miR-30b, miR-124, miR-145, miR-155, miR-182, miR-320b, miR-342, miR-370, miR-451b, and miR-574 were also reported to be involved with atherosclerosis development [92,94,105,106,108].

MiR-484 plays a role in cardiomyocyte apoptosis [95], and its direct interaction with miR-361 has implications for cardiac diseases such as myocardial infarction [104]. Reduced expression of miR-125b-5p in both humans and mice is related to cardiac ischemia and has been proposed as a diagnostic biomarker as well as a biomarker of progression to heart failure [108].

#### 5.2.2. Cardiac Hypertrophy and Fibrosis

MiR-21 is a key molecule involved in cardiac hypertrophy that promotes angiogenesis and cardiomyocyte survival post-myocardial infarction [98] and inhibits apoptosis and inflammation [98]. miR-21 is mainly expressed in cardiac macrophages that control myocardial fibrosis through intercellular signals to fibroblasts [107]. Overexpression of miR-21 triggered by hypoxia in murine hearts has been linked to pathological cell growth and cellular stress [96]. Elevated expression of miR-21 has also been observed in cardiac tissue from patients with heart failure. However, genetic deletion of miR-21 does not alter the pathological cardiac response to pressure overload [109,110].

MiR-133 inhibits the AKT/mTOR pathway and the beta-adrenergic receptor (β-AR), inhibiting activation cascades that could lead to atrophy, apoptosis, and autophagy [111]. Hypertrophy and fibrosis are also reduced by overexpressed miR-1 [112].

In addition, miR-127-3p has been identified as a pro-fibrogenic agent and was upregulated in rat hearts during cardiac fibrosis. When overexpressed, miR-127-3p increased fibrogenic differentiation and proliferation, and its inhibition suppressed fibroblast activation [94].

MiR-208 is related to various cardiac processes, including increased cardiac fibrosis via THRAP-1 (Thyroid-Hormone-Receptor-Associated Protein 1) inhibition, right ventricular hypertrophy by inhibiting the Mef2 axis, and negative regulation of the SOX6 (SRY-Box Transcription Factor 6) gene associated with cardiac hypertrophy [100]. miR208a has been described as specifically expressed in cardiac tissue promoting cardiac hypertrophy in animal models [100].

Bostjancic et al. [111] demonstrated upregulation of cardiac miR-208 and the downregulation of miR-1 and miR-133a in human autopsy samples of infarcted heart tissue [112,113]. Some patterns of miRNA expression observed in myocardium infarction hearts were similar to those observed in fetal hearts [108,114,115,116].

#### 5.2.3. Heart Failure

Heart failure (HF) is a clinical syndrome with symptoms including dyspnea, ankle swelling, and fatigue (European Society of Cardiology, 2016). HF is characterized by reduced ability of the heart to deliver blood at a rate adequate to meet tissues requirements [117]. Symptoms—such as pulmonary crackles, peripheral oedema, and increased pressure in the jugular veins—result in increased intracardiac pressure or decreased cardiac output at rest or during stress [118]. Several etiologies and specific risk factors and co-morbidities are involved in HF, with miRNAs playing a key role in those processes [100].

miR-21-5p plays an important role in the proliferation and apoptosis of vascular smooth muscle cells and cardiac cells, and also affects the function of cardiac fibroblasts [98]. miRNA-21-enriched exosomes are secreted by cardiac fibroblasts, which mediate cardiomyocyte hypertrophy through a paracrine signaling mechanism [92]. In the cells of a failing heart, high MiR-21 levels inhibit the Sprouty homolog 1 (Spry1) protein, thus increasing ERK-MAP kinase (extracellular-signal-regulated kinase–mitogen-activated protein kinase). This mechanism regulates the extent of interstitial fibrosis and cardiac hypertrophy that often leads to heart failure [92].

Concentrations of miR-1 or miR-499 are abundant in the blood only when the coronary arteries are affected [95,111]. Thus, circulating miR-1 is used as an independent predictive indicator of LV remodeling after ST-elevation myocardial infarction (STEMI) [119]. Another promising biomarker of acute NSTEMI (non-ST-elevation myocardial infarction) is miR-499-5p. Elevated miR-499 levels were associated with atrial fibrillation, which is a leading cause of HF [93].

The miR-29 family (miR-29a/b/c) exert physiological regulatory effects in cardiomyocytes, aortic and myocardial tissue, vascular endothelial cells, and cardiac metabolism. They also play various regulatory functions in the pathophysiological mechanisms of cardiomyopathies, atrial fibrillation, myocardial fibrosis, atherosclerosis, and heart failure. Expression of the miR-29 family tends to be reduced in the affected cardiac tissue. On the other hand, high levels of miR-29b reduced collagen expression [103]. Thus, miR-29 family members are considered anti-fibrotic markers [103].

## 6. Stress

Environmental stress has been identified as a relevant cardiovascular risk factor [120,121]. Cardiovascular pressure/volume homeostasis depends on neurohormonal systems including the renin–angiotensin–aldosterone (RAAS) and sympathetic nervous systems together with the cardiac natriuretic peptides. Catecholamines released by the sympathetic nervous system–adrenal gland medulla, coupling to cardiac β-adrenergic receptors, increase cardiac output. However, during chronic stress the excessive catecholamine stimulation together with glucocorticoids released by the hypothalamus–hypophysis–adrenal gland cortex promote changes in the cardiac β-adrenergic receptor population [122,123], with consequences for heart function [124].

We have reported that, in an experimental model of stress, cardiac expression of several genes codifying proteins of the β-adrenergic receptor and glucocorticoid receptor signaling pathways were altered [125]. The repertoire of genes with significant differences in expression confirms that environmental stress may cause phenotypic changes in cardiac cells [125]. Indeed, microarray data have shown the participation of the following miRNAs in the modulation of the cardiac function under stress: miR-331-5p, miR-331-3p, miR-127-3p, miR-125b-5p, miR-191-5p, and miR-30c-5p [124].

miR-208 regulates the expression of genes associated with cardiac function, such as the Myosin Heavy Chain β (β-MHC) gene, contributing to cardiac remodeling aimed at enhancing performance [119]. MiR-208 is encoded within the introns of various myosin genes. When encoded within a fast myosin gene (β-MHC), it regulates the expression of miR-499 and miR-208b; when encoded within a slow myosin gene (α-MHC), it forms feedback loops that regulate miRNA levels and, consequently, muscle contraction in response to physiological changes. MiR-208 has been reported to be activated when the heart is exposed to elevated blood pressure or workload, triggering a series of adaptive responses.

## 7. The Clinical Potential of miRNAs: Diagnostic, Prognostic, and Therapeutic Implications

Currently, heart disease diagnosis is mainly based on non-imaging biomarkers, such as cardiac troponin, both I and T forms, C-reactive protein and high-sensitivity C-reactive protein, creatine kinase, natriuretic brain peptide (BNP), and NT- pro BNP, in addition to coronary angiography. Although those protein evaluation techniques have the advantages of being non-invasive and without radiation exposure, they present some limitations. Despite being highly specific, troponins detect myocardial injury only after necrosis has occurred; creatine kinase is less specific than troponins; BNP and hs-BNP indicate heart failure but are influenced by obesity, age, and kidney function; and C-reactive protein is a marker of inflammation with low specificity. As a consequence, those available protein-based biomarkers for identifying cardiac diseases frequently provide false-positive results that lead to wrong diagnosis and treatment [126].

As an alternative, coronary angiography allows the anatomical and functional assessment of the coronary artery [126]. However, technical factors, the complexity of coronary anatomy, and plaque configuration may result in misdiagnosis [127], and the use of local anesthesia and contrast material may pose a risk of health-related complications to patients [127].

Hence, the development of microRNA evaluation techniques offers an additional alternative to detect heart diseases [126]. As compared to the actual biomarkers, miRNAs detect changes before necrosis, enabling early diagnosis, whereas troponins detect changes only after necrosis; miRNAs can be more specific for different types of cardiac injury. In heart failure patients, miRNA-423-5p is highly expressed in the blood irrespective of age and gender and might be a sensitive biomarker for heart diseases [127]. Moreover, plasma miRNA-423-5p is elevated in dilated cardiomyopathy patients and has a positive correlation with the N-terminal pro-brain natriuretic peptide [128]. A recent study showed that the serum of coronary artery disease patients had reduced circulating miRNA-126 and miRNA-145 [129]. It has been shown that miRNA-208 are upregulated and miRNA-1 and miRNA-133a/b are downregulated in cardiac tissue samples from myocardium infarction patients when compared to normal controls [130].

Thus, some researchers believe that miRNAs could be used as highly sensitive early diagnostic biomarkers and therapeutic targets for many cardiovascular diseases such as atherosclerosis, acute myocardial infarction, heart failure, hypertension, and stroke [105,127,129]. Nevertheless, some miRNAs lack specificity, since miRNAs are expressed differently in various diseased states and tissues [120]. Population differences, gender, and comorbidities are additional confounding factors to be taken into consideration, as they were reported to affect the profiles of circulating miRNA [130].

MiR-208a, for example, is one of the cardiac miRNAs which have been relatively well investigated. Yet, our understanding of this miRNA appears to still be rudimentary. Numerous topics need to be addressed, such as its biological functions and the corresponding mechanisms, its multiplicity, the broad range of downstream targets, and the complex regulatory network(s), which remain very elusive. This and many other miRNAs hold great potential as therapeutic targets; however, extensive studies and analyses are required prior to full clinical application.

Therefore, there is still a lack of research focusing on identifying and validating miRNAs as heart disease diagnostic markers in clinical practice.

So, deciphering the precise roles of specific miRNAs actions in physiological and pathological contexts is still a big challenge. Thus, the actual challenge is to establish standard protocols that optimize and simplify the work of miRNA isolation and its expression level estimation.

## 8. Future Perspectives and Conclusion

The newly revealed role played by miRNAs as mRNA modulators opened a new perspective regarding diagnosis and therapeutics of many diseases, including cardiovascular diseases.

However, before their use as therapeutic agents or marker of some disease, it must be taken into account that the regulation of mRNA expression by miRNAs depends on many factors. Usually there is no specific correlation between the level of miRNA expression and its action, since its expression can be intermittently altered in pathophysiological contexts and its activity and availability can also be regulated [100].

Another major drawback in miRNA research concerns the difficulty of linking molecular knowledge to our understanding of miRNA function at the cellular and intercellular levels. An integrative view of spatial and temporal aspects of miRNA function under homeostatic and stressed conditions is not available yet.

This demand is more prevalent in cardiac diseases since they are the main cause of death worldwide. This research should facilitate the design of new therapeutic approaches based on miRNA targeting molecular mechanisms underlying complex diseases. Their use as therapeutic tools is on the horizon.
